# Patulin contamination in apple products marketed in Shiraz, Southern Iran

**DOI:** 10.29252/cmm.3.4.32

**Published:** 2017-12

**Authors:** Ali Poostforoushfard, Ahmad. R Pishgar, Enayat Berizi, Hasti Nouraei, Zahra Sobhani, Rohallah Mirzaie, Kamiar Zomorodian

**Affiliations:** 1 Vice Chancellery for Research Affairs, Shiraz University of Medical Sciences, Shiraz, Iran; 2 Student Research Committee, Shiraz University of Medical Sciences, Shiraz, Iran; 3 Department of Food Hygiene and Quality Control, School of Nutrition and Food Sciences, Shiraz University of Medical Sciences, Shiraz, Iran; 4 Basic Sciences in Infectious Diseases Research Center, Shiraz University of Medical Sciences, Shiraz, Iran; 5 Food and Drug Department, School of Pharmacy, Shiraz University of Medical Sciences, Shiraz, Iran; 6 Medicinal Plants Processing Research Center, Department of Medical Mycology and Parasitology, School of Medicine, Shiraz University of Medical Sciences, Shiraz, Iran

**Keywords:** Apple juice, Mycotoxin, Patulin

## Abstract

**Background and Purpose:**

Patulin is one of the important mycotoxins, produced by a wide range of molds, including *Penicillium*, *Aspergillus,* and *Bysochlamys. *Patulin is mainly found in the rotten parts of fruits and vegetables, such as apples, pears, peach, apricots, and grapes. Currently, the Codex Alimentarius and Food and Drug Administration have recommended a maximum level of 50 µg/L patulin for apple products. The purpose of this study was to investigate patulin contamination of apple juice and cans in 75 samples collected from 15 manufacturers in Shiraz, southern Iran.

**Materials and Methods:**

The detection of patulin was accomplished using a high-performance liquid chromatography with an ultraviolet detector.

**Results:**

A total of 38 apple juice samples (53%) and 17 apple cans (45%) were contaminated with patulin. Overall 50% and 3% of the apple juice and apple cans samples had a patulin level of > 3 µg/L.

**Conclusion:**

Although the maximum level of patulin in our samples was considerably lower than the permitted level established by the European Union (i.e., 50 µg/L), the high incidence of this mycotoxin in our samples should be lessen by improving their good manufacturing practice.

## Introduction

Mycotoxins are secondary abiotic hazard metabolite produced by fungi in the contaminated foods [1, 2]. The toxins may form on fields or during the storage or even processing of the foods [3]. Patulin (4-hydroxy-4H-furo[3,2-c]pyran-2(6H)-one), is one of the important mycotoxins, produced by a wide range of molds, in particular *Penicillium*, *Aspergillus,* and *Bysochlamys*, among which *Penicillium expansum* is the most isolated species [4, 5]. 

Patulin is mainly found in the rotten parts of fruits and vegetables, such as apples, pears, peaches, apricots, and grapes [6]. Although this heat-resistance toxin was initially studied as a potential new antibiotic, different studies have demonstrated its immunotoxic [7], genotoxic [8], embryotoxic, and neurotoxic properties [9]. Patulin has been recognized as a health-threatening substance in several countries. Patulin is reported more frequently in apple products among other different fruit products [1]. Currently, the Alimentarius and Food and Drug Administration have recommended a maximum level of 50 µg/L patulin for apple products [10, 11]. 

Accordingly, the European Union in the Commission Regulation 1425/2003 has recommended the patulin levels of < 50, 25, and 10 µg/L for apple juice, beverage containing apple juice, and solid apple products, respectively [12, 13]. The level of patulin in apple products has been reported in several countries. In a study conducted in Tunisia, the concentration of patulin in 35% of the samples was over 50 µg/L [14]. In some other studies performed in Spain [15] and Japan [16], the apple products were reported to be contaminated with patulin. In Iran, Karimi et al. observed the concentration level of ≥ 50 µg/L patulin in 10% of the samples [11]. 

There are limited number of studies investigating this mycotoxin in Iran. Regarding this and considering the toxic effects of patulin on people’s health, especially children, the present study was conducted with the aim of estimating the level of patulin in apple juice and cans of different brands. 

## Materials and Methods

***2.1. Sample collection***

This study was conducted on a total of 75 samples, including 38 and 37 samples of apple juice and apple cans, respectively, which were randomly purchased from the retail markets during 2016. These samples were produced by 15 Iranian manufacturers.

***2.2. Reagents and Materials***

Patulin (with purity of ≥ 99%) and hydroxymethylfurfural standards were supplied from the Sigma-Aldrich Corporation (USA). Water and acetonitrile supplied of high-performance liquid chromatography (HPLC) grade and other chemicals, such as acetone, ethanol, methanol, chloroform, carbon tetrachloride, dichloromethane, carbon disulfide, ethyl acetate, and sodium chloride, were obtained from the Merck Company (Darmstadt, Germany). The stock solution of patulin was 10 mg/L. Patulin and standard solutions were prepared by dilution in ethanol. The stock solution was stored at -20°C. The apple juice samples were collected from different supermarkets (Shiraz, Iran).

***2.3. Sample Preparation***

The samples were stored at ambient temperature before further use. Once opened, they were stored in specific food containers at 4°C and analyzed within 5 days. The fresh juice was centrifuged at 3,500 rpm for 15 min, the supernatant was then filtered through a 0.45-μm membrane filter. Afterwards, the filtrate was diluted at 1:4 ratios with deionized water and used for dispersive liquid-liquid microextraction (DLLME) procedure [1].

***2.4. Instrumentation***

The HPLC system equipped with an auto-sampler (Waters 717), an HPLC pump (Waters 1525), and a dual λ absorbance ultraviolet detector (Waters 2487) were used for the analysis. A chromolith HPLC column (15 cm, Agilent) was selected for the measurement. A mixture of water and acetonitrile (97:3) at a flow rate of 1 mL/min was utilized as the mobile phase in isocratic elution mode. The detection was performed at a wavelength of 276 nm. The separation of the dispersive phase was performed using a centrifuge model, namely Tlettich universal 320.

***2.5. Sample treatment***

For the implementation of DLLME, under optimum conditions, 5.00 mL blank apple juice was spiked with 5 μL of 50 ppb patulin in a 10-mL glass test tube. The extraction solvent contained a mixture of 1.0 mL acetonitrile as the disperser and 250 μL ethyl acetate: chloroform (190 μL: 60 μL). The extraction solvent was rapidly injected into the sample solution by a glass syringe. 

After forming a cloudy solution, the extraction was centrifuged at 4,000 rpm for 3 min; subsequently, chloroform and ethyl acetate were collected from the bottom of a conical test tube. The deposited phase was completely transferred to another test tube and evaporated to dryness. The residue was reconstituted in 1000 μL HPLC-grade acetonitrile, and 100 μL was injected into the HPLC system.

## Results

The concentration levels of patulin in the samples of apple juice and cans are tabulated in Table 1. According to the results, a total of 38 apple juice samples (53%) were contaminated with patulin; however, in most of the cases, the concentration was mostly lower than the standard limit of Codex Alimentarius (i.e., <50 µg/mL for apple products). The concentration of patulin was above 3 µg/mL only in 19 (50%) apple juice samples. The maximum concentration of patulin in the apple juice samples was recorded as 39.5 µg/mL.

Contamination with patulin was substantially lower in the apple cans in comparison with that in the apple juice samples. As the results indicated, no evidence of patulin contamination was detected in 21 (55%) apple cans; in addition, the concentration of patulin was lower than 3 µg/mL in 37 (97%) cases. Furthermore, the maximum level of this toxin in apple cans was 34.8 µg/mL.

The results of the present study revealed the widespread contamination of the apple juice produced in Shiraz with patulin; nonetheless, this contamination was not at a high level. Patulin is one of the important contaminants of apple and other fruit products. This is a secondary toxic metabolite produced by a wide range of fungi, such as *Aspergillus* and *Penicillium*. Mold growth usually leads to the production of patulin on the damaged surface of the apples [17, 18]. Patulin is currently considered as a genotoxic, immunotoxic, neurotoxic, carcinogenic, and immunosuppressive agent [19].

**Table 1:** Concentration of patulin in the apple juice and cans samples

		**Patulin (µg/mL)**			
**Sample type**	**No.**	**Negative**	**≤3 µg/mL**	**3-50 µg/mL**	**Range**
Apple juice	38	18 (47%)	1 (3%)	19 (50%)	0-39.5
Apple cans	38	21 (55%)	16 (42%)	1 (3%)	0-34.8
Total	76	39 (51%)	17 (22%)	20 (27%)	0-39.5

In the recent years, many studies have been performed to evaluate the level of patulin in apple products and other fruits. Studies in different locations have reported dissimilar patulin concentrations. These concentration levels were 2335, 0.7-38.8, 0.9-11.8, 0-167, and 0.7 -101.90 µg/L in South Africa (in rotten fractions of apples), Belgium (in apple juice), Greece, Tunisia, and Romania, respectively [14, 20-23]. In the recent years, few studies in Iran have been targeted toward the investigation of patulin level in the apple products. 

In this regard, Cheraghali et al. demonstrated that 33% of apple juice samples were contaminated with patulin at the concentration level of > 50 µg/L and a maximum level of 285 µg/L. In addition, they observed that patulin level was higher than the authorized level in 56% of apple juice concentrate samples [6]. Furthermore, Karimi et al. investigated the presence of patulin in 58 apple juice samples in Mashhad province, Iran. The demonstrated that 54 cases were contaminated with patulin at the concentration range of 10.1-121.8 µg/L. They observed that the level of patulin was higher than 50 µg/L [11] in 10% of the samples. 

In another study conducted by Jalali et al. on 150 apple juice samples collected from the Southwest reign of Iran, the level of contamination ranged within 50-106.01 µg/L in 13.3% of the samples with the mean of 26.92 µg/L [24]. Farhadi and Maleki reported the patulin concentration range of 8-40 µg/L in apple juice and apple juice concentrate samples [25]. Moreover, Forouzan and Madadlou (2014) investigated 72 apple juice samples produced in West Azerbaijan province, Iran, and reported that all of the samples were contaminated with patulin at concentrations ranging 29.58-151.2 µg/L. They found that patulin concentration was higher than 50 µg/L in 29.16% of the samples [26]. 

Rahimi and Rezapoor (2015) conducted a survey to determine patulin level in 161 samples of fruit juices, including apple, pineapple, pear, peach, pomegranate, as well as white and red grape juices. In the mentioned study, 16.1% of the samples were contaminated (range: 5-190.7 µg/kg), and 2.5% of the apple juice samples were contaminated at concentration higher than 50 µg/L [27].

In the present study, 38 apple juice samples and 38 apple cans were studied for patulin contamination. The results showed that the level of patulin was higher than 3 µg/mL in 50% of the apple juice samples and 3% of the apple cans. The maximum levels of patulin in the apple juice and apple cans samples were 39.5 and 34.8 µg/mL, respectively. The maximum level of patulin in our samples was considerably lower than the permitted level established by the European Union (i.e., 50 µg/L). 

The observation of patulin concentration of < 3 µg/L in the majority of the samples indicated the importance of implementing effective prevention strategy in all stages of harvesting, processing, and storing of this fruit product. Accordingly, the efficient removal of patulin during processing and post-production detoxification were recommended. In conclusion, the high incidence of patulin in the samples should be lessen by improving hygienic storage, sorting and trimming rotten fruits, filtration through activated charcoal, and pasteurization.
